# Successful detection of covert paroxysmal atrial fibrillation due to insertable cardiac monitor in embolic stroke of undetermined source in a patient with situs inversus totalis

**DOI:** 10.1002/ccr3.3826

**Published:** 2021-01-19

**Authors:** Yuhei Kasai, Sandeep Shakya, Kotaro Miyaji, Junji Kanda

**Affiliations:** ^1^ Department of Cardiology Asahi General Hospital Chiba Japan

**Keywords:** covert paroxysmal atrial fibrillation, embolic stroke of undetermined source, insertable cardiac monitor, situs inversus totalis

## Abstract

Insertable cardiac monitors in patients with situs inversus totalis can detect atrial arrhythmia as a cause of embolic stroke. It is important to premap the position of ICM in order to clearly visualize the P wave.

## Graphical abstract

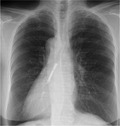

## 1 INTRODUCTION

We describe a case of ICM implantation in a 74‐year‐old woman with SIT and ESUS for detection of AF. ICM implantation was performed successfully without any complications. AF was detected at 28 days after ICM insertion. ICMs can be safely implanted in patients with SIT, allowing successful detection of AF.

Approximately, 25% of all ischemic strokes are cryptogenic.^1^ Of these, a clinical entity of embolic stroke of undetermined source (ESUS) was recently established. ESUS is defined as a nonlacunar brain infarction without a proximal arterial steno‐occlusive lesion or cardioembolic source. Thus, the pathogenesis of ESUS is varied and includes covert paroxysmal atrial fibrillation (CPAF), aorto/arteriogenic embolism, paradoxical embolism, and cancer‐related embolism. CPAF is a major cause of ESUS.^1^ However, detecting CPAF during hospitalization is often difficult, even with continuous electrocardiographic (ECG) monitoring.

Insertable cardiac monitors (ICMs) combined with remote monitoring have been used in ESUS patients with mild symptoms (modified Rankin scale from 0 to 2) to detect CPAF and to prevent recurrent ischemic stroke. ICMs are usually implanted into the left margin of the sternum, from the 3rd to the 6th intercostal space parallel to the cardiac shadow, using the provided insertion tools.^2^ Because these tools are very simple and easy to use, complications of ICM (eg, bleeding or infection) are very rare. Previous report describes insertion of ICM in pediatric population to detect ventricular arrhythmia for syncope.^3^ However, to our knowledge, there are no reports of ICM implantation in patients with situs inversus totalis (SIT) to detect atrial arrhythmia in ESUS patient. Herein, we describe ICM implantation for detection of CPAF as an embolic source of ischemic stroke in a patient with SIT.

## 2 CASE REPORT

**FIGURE 1:**
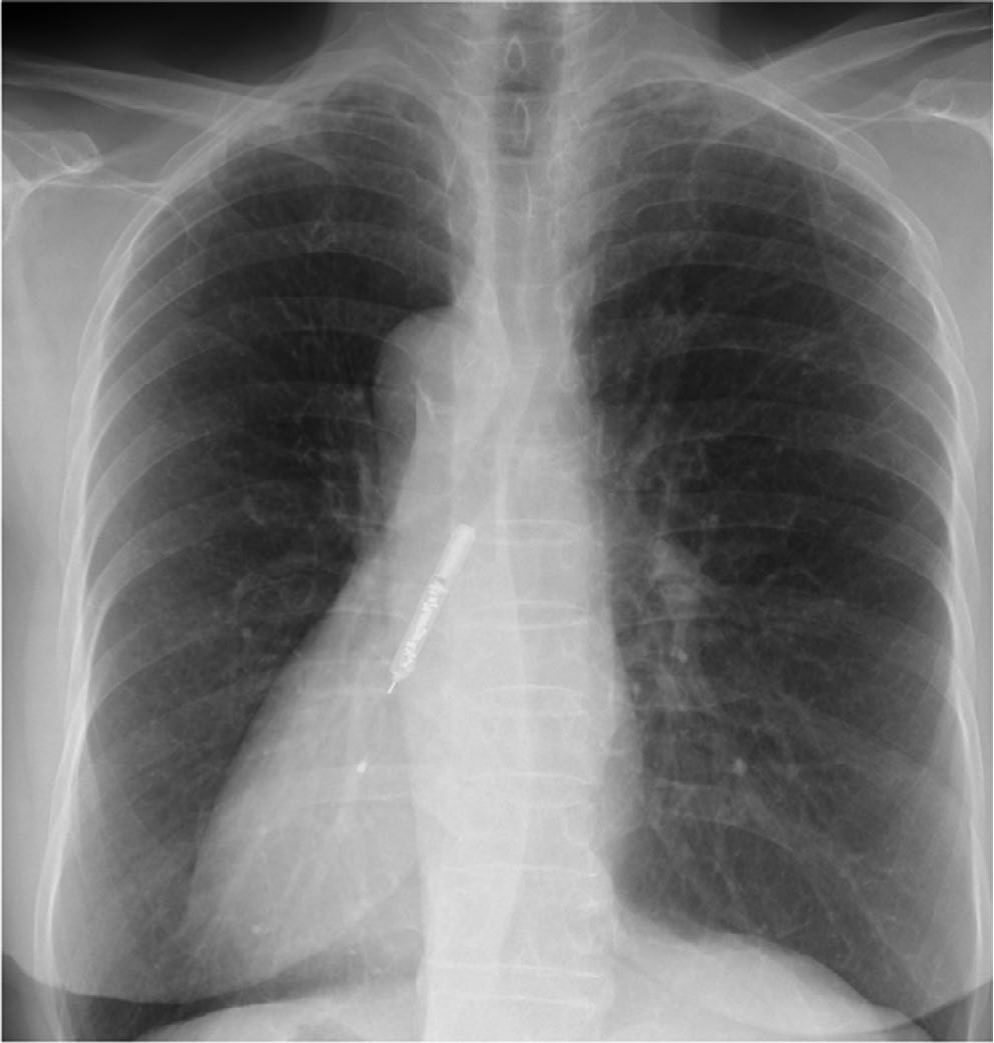
Chest radiograph shows cardiac apex points to the right, indicating situs inversus totalis. Insertable cardiac monitor can be seen in the right margin of the 3rd to 6th intercostal sternum along the long axis shadow of the heart

A 75‐year‐old woman with sudden right hemiparesis was admitted to our hospital with a diagnosis of acute ischemic stroke. She was treated with intravenous tissue plasminogen activator and thrombectomy in the left M2 segment of the middle cerebral artery, after which her symptoms improved remarkably (modified Rankin scale 0). The patient met the criteria of ESUS, and a thorough examination was performed to determine the cause. Transesophageal echocardiography showed minor plaque formation in the aortic arch (a potential cause of aortogenic embolism) and absence of a patent foramen ovale (a potential cause of paradoxical embolism). The patient had the HAVOC score of 4 (hypertension, age).^4^ Although CPAF was not identified by telemetry at admission, it was suspected and we decided to implant an ICM device. However, SIT was observed by chest X‐ray. Thus, the ICM device was implanted into the right chest in a symmetrical position (Figure 1).

We used fluoroscopy to detect the precise location of the heart. In order to detect the P waves clearly in ICM, we did a simple premapping with surface ECG. During premapping, ECG was recorded at the angle of 0 degrees, 30 degrees, 60 degrees, and 90 degrees in 3rd and 4th intercoastal space respectively, and the site was chosen with the highest QRS/T ratio and relatively higher P wave. ECG at the angle of 60 degrees in 3rd intercoastal space had the best ECG, and an ICM (BIOMONITOR IIITM; Biotronik) was then inserted into the right margin of the 3rd intercostal sternum along the long axis shadow of the heart at 60 degrees angle using the provided tools. The procedure duration was 5 minutes, and the fluoroscopy time was 15 seconds. There were no complications during the procedure.

**FIGURE 2:**
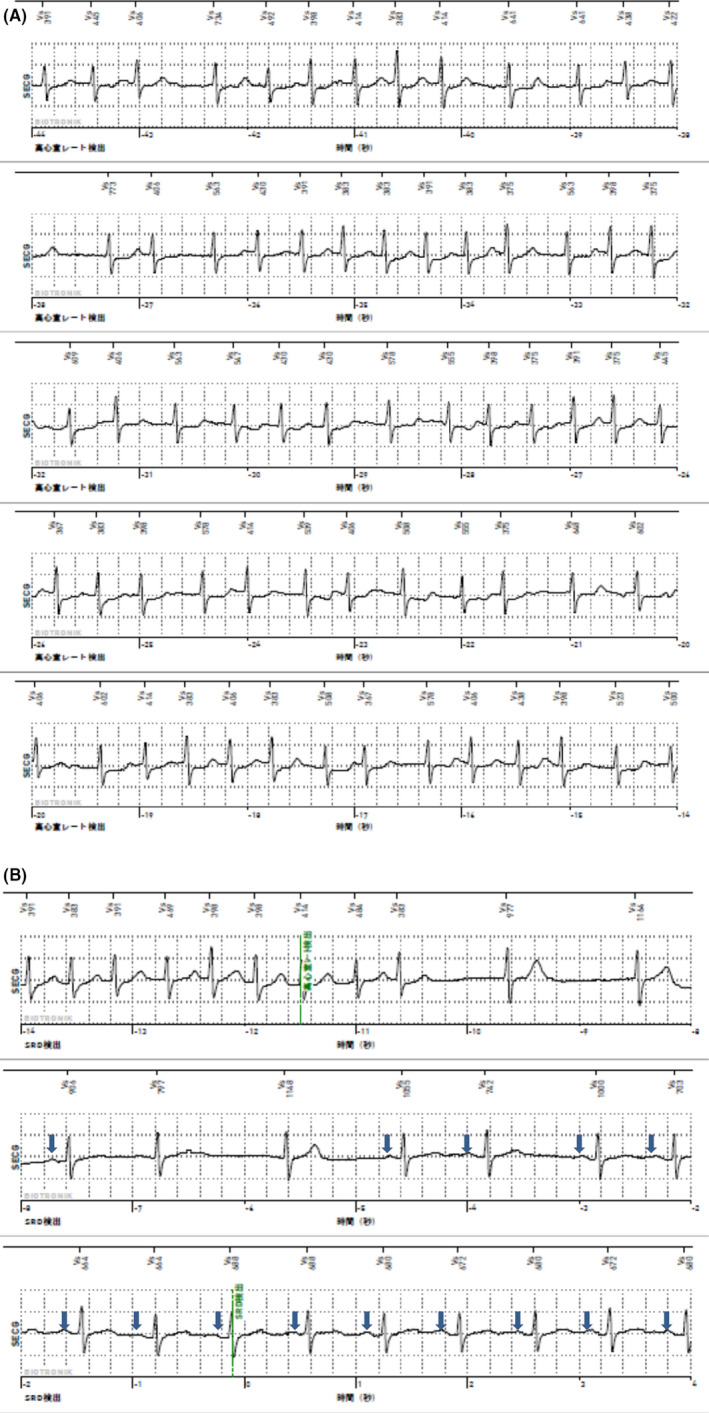
A, Subcutaneous ECG showing atrial fibrillation. B, It shows the sudden drop rate alarm and the feature unique to the BIOMONITOR III™ device. P wave (arrow) can be clearly seen during the sinus rhythm

The first AF episode was successfully detected at 28 days after ICM insertion (Figure 2A). Within 6 months, the AF lasting for more than 1 minute was detected 28 times and AFL was detected 15 times in total. Figure 2B shows the sudden drop rate alarm, the feature unique to the BIOMONITOR III™ device. It shows the moment when rhythm changed from AF to sinus rhythm. P wave can be clearly seen during the sinus rhythm. AF was then detected on a number of occasions.

Aspirin was used initially for prevention of recurrent ischemic stroke. However, we changed to edoxaban after CPAF was detected.

## 3 DISCUSSION

To our knowledge, this is the first report of CPAF detection in an ESUS patient with situs inversus totalis. The detection rate of CPAF in ESUS patients using ICM is 30%.^5^

However, because the incidence of SIT is very low in the general population^6^ and because the heart and the conduction system are located on the right side of the body, and the safety and efficacy of ICM in these patients remain unclear. In our experience, ICM insertion into the right margin of the sternum along the heart shadow using fluoroscopy is safe and effective for detecting CPAF.

There are three types of ICM devices used in Japan. In the present case, we used the BIOMONITOR III, a novel ICM device that combines a long sensing vector with a miniaturized profile. Previous studies have described visible P waves in >80% of cases.^2^ In our case, there was no P wave in the subcutaneous ECG during AF compared with that during sinus rhythm (Figure 2A, B). In the subcutaneous ECG recorded by the sudden drop rate alarm, the P wave was not visible during AF, but appeared when the system returned to sinus rhythm. These findings provided a clear diagnosis of AF rather than sinus tachycardia, and the patient's medicine was changed from aspirin to edoxaban. There are several case reports on the feasibility and safety of catheter ablation for AF with SIT.^7^ Thus, if our patient shows drug‐refractory AF, we will consider catheter ablation.

## 4 CONCLUSION

Insertable cardiac monitors (eg, the BIOMONITOR III™) implantation can be performed safely in patients with situs inversus totalis and can be used to detect CPAF.

## CONFLICT OF INTEREST

None declared.

## AUTHOR CONTRIBUTIONS

YK: wrote the manuscript. SS: participated in this ICM implantation and supervised the writing of the manuscript. KM: supervised the writing of the manuscript. JK: supervised the manuscript. All authors: read and approved the final manuscript.

## ETHICAL APPROVAL

The enrolled patient provided written informed consent. The examination was made in accordance with the approved principles. All the preparations and the equipment used are officially certified for the clinical use.

## Data Availability

Data are available on request.
